# The Role of Parasitoid Wasps, *Ixodiphagus* spp. (Hymenoptera: Encyrtidae), in Tick Control

**DOI:** 10.3390/pathogens12050676

**Published:** 2023-05-03

**Authors:** Rafael Antonio Nascimento Ramos, Lucia Oliveira de Macedo, Marcos Antônio Bezerra-Santos, Gílcia Aparecida de Carvalho, Guilherme Gomes Verocai, Domenico Otranto

**Affiliations:** 1 Laboratory of Parasitology, Federal University of the Agreste of Pernambuco, Garanhuns 55292-270, PE, Brazil; luciamacedo162@gmail.com (L.O.d.M.); gilcia.carvalho@ufape.edu.br (G.A.d.C.); 2 Department of Veterinary Medicine, University of Bari, Valenzano, 70121 Bari, Italy; marcos.bezerrasantos@uniba.it (M.A.B.-S.); domenico.otranto@uniba.it (D.O.); 3 Department of Veterinary Pathobiology, School of Veterinary Medicine & Biomedical Sciences, Texas A&M University, College Station, TX 77843, USA; gverocai@cvm.tamu.edu; 4 Faculty of Veterinary Sciences, Bu-Ali Sina University, Hamedan 65174, Iran

**Keywords:** *Ixodiphagus hookeri*, biological control, ixodid, argasid, vectors, hymenopteran

## Abstract

Species of *Ixodiphagus* (Hymenoptera: Encyrtidae) are parasitoid wasps whose immature forms develop inside ixodid and argasid ticks (Acari: Ixodida). Following oviposition by adult female wasps into the idiosoma of ticks, larvae hatch and start feeding on their internal contents, eventually emerging as adult wasps from the body of the dead ticks. Species of *Ixodiphagus* have been reported as parasitoids of 21 species of ticks distributed across 7 genera. There are at least ten species described in the genus, with *Ixodiphagus hookeri* being the most studied as an agent for biological control of ticks. Although attempts of tick control by means of this parasitoid largely failed, in a small-scale study 150,000 specimens of *I. hookeri* were released over a 1-year period in a pasture where a small cattle population was kept, resulting in an overall reduction in the number of *Amblyomma variegatum* ticks per animal. This review discusses current scientific information about *Ixodiphagus* spp., focusing on the role of this parasitoid in the control of ticks. The interactions between these wasps and the ticks’ population are also discussed, focusing on the many biological and logistical challenges, with limitations of this control method for reducing tick populations under natural conditions.

## 1. Introduction

Species within the genus *Ixodiphagus* (Hymenoptera: Encyrtidae) are natural parasitoid wasps of ticks (Acari: Ixodida) [1], which were first described more than a century ago, in *Haemaphysalis leporispalustris* from Texas, United States (USA) [2]. The etymology of the genus name *Ixodiphagus* (from Greek ixod = tick and phage = eater) alludes to its parasitoid behavior. After its first description, other species of “tick eaters” within this genus were formally described worldwide [3,4,5,6]. 

Currently, at least ten species of these parasitoids are considered valid, namely *Ixodiphagus texanus* Howard, 1907; *Ixodiphagus hookeri* Howard, 1908; *Ixodiphagus mysorensis* Mani, 1941; *Ixodiphagus hirtus* Nikolskava, 1950; *Ixodiphagus theilerae* Fielder, 1953; *Ixodiphagus biroi* Erdos, 1956; *Ixodiphagus sagarensis* Geevarghese, 1977; *Ixodiphagus taiaroaensis* Heath and Cane, 2010; *Ixodiphagus sureshani* Hayat and Islam, 2011; and *Ixodiphagus aethes* Hayat and Veenakumari, 2015. These insects are small, generally measuring less than 1 cm in length, blackish in color, and exhibiting the typical appearance of members of the superfamily Chalcidoidea, and display similar biological and ecological features [7]. 

Despite being known for over a century, many knowledge gaps remain about the biology of these parasitoid wasps, with most information limited to *I. hookeri* [5,8]. The life cycle of these wasps starts when gravid females lay eggs inside the tick’s body. After an incubation period, the larvae hatch and feed on the internal content of the tick [7]. Approximately 30–57 days after oviposition, new adult male and female wasps emerge from the dead tick, mating and continuing their life cycle [9]. Based on this life cycle, the use of *Ixodiphagus* spp. as an agent for biological control of ticks has inspired the interest of the scientific community [10]. In addition, populations of *I. hookeri* may have different developmental times, parasitism rates, and host preferences according to the geographical area of occurrence [10], which may explain the failure, or the limited efficacy, of these wasps in the control of ticks in field studies [11,12]. 

For many decades the use of acaricide drugs for the control of ticks on animal hosts has been extensively applied worldwide [13], with some classes of drugs (e.g., organophosphates, pyrethroids, amidines, and macrocyclic lactones) widely used. The excessive and/or incorrect use of these compounds, through metabolic detoxification or changes in the sensitivity of the target site of drug action [14], generates the appearance of acaricide resistance in certain tick species and populations [15]. Because of ticks’ major economic impact on livestock production, resistance to acaricide drugs has been considered one of the most significant threats to veterinary medicine in the last decade [16]. With increased concerns about the use of chemicals and reports of their lack of efficacy [17], the interest in alternative biological control methods increased [18,19,20]. Additionally, the implementation of biological control strategies could mitigate direct impacts of ticks and potentially reduce transmission of certain tick-borne pathogens. For instance, it has been demonstrated that *Ixodes scapularis* parasitized by *I. hookeri* on Naushon Island, Massachusetts, USA, did not carry *Borrelia burgdorferi* sensu lato and rarely carried *Babesia microti*, despite the presence of these zoonotic pathogens in uninfected ticks in the same area [21]. More recently, the presence of *Arsenophonus nasoniae* and *Rickettsiae* infections in *Ixodes ricinus* were attributed to the presence of *I. hookeri* [22]. Therefore, in this review we discussed the interactions between these wasps and tick populations, focusing on the limitations of this approach under natural conditions. 

## 2. Biology of *Ixodiphagus* spp. and Geographic Distribution

**Figure 1:**
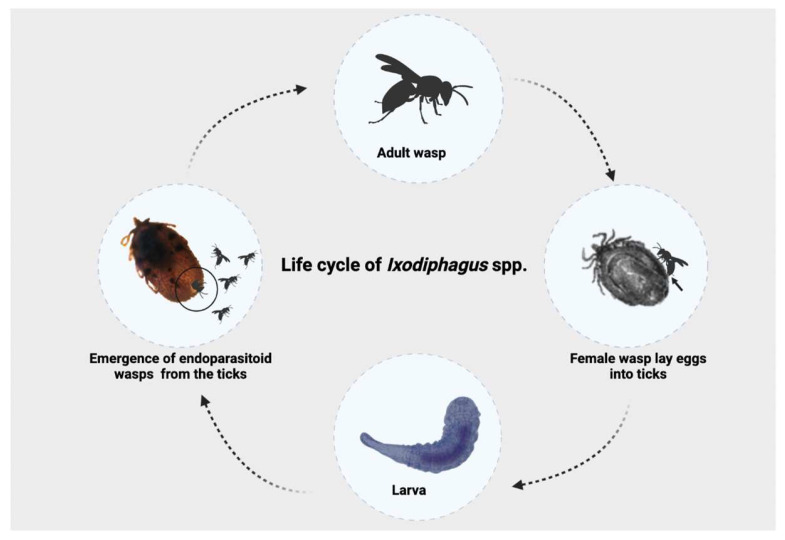
Life cycle of *Ixodiphagus* spp.

**Figure 2:**
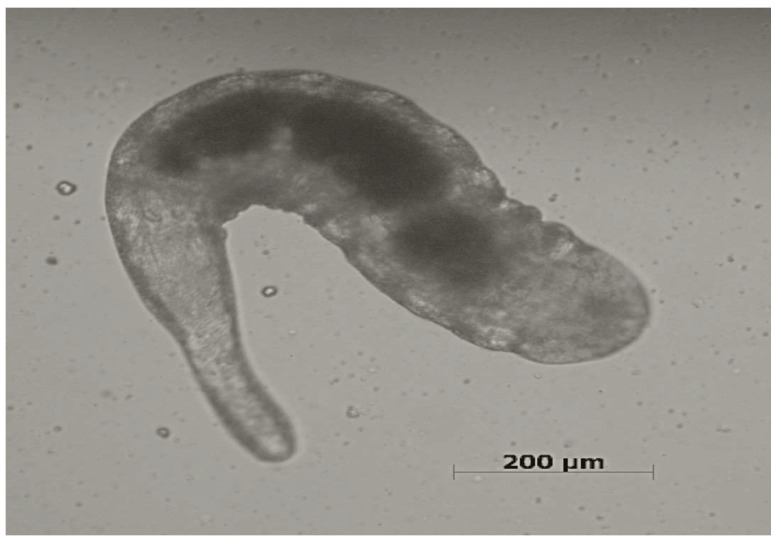
*Ixodiphagus* sp. larva in a *Rhipicephalus sanguineus* s.l. tick (Scale bar = 200 μm).

Information on the biology of *Ixodiphagus* species is insufficient and mainly limited to experimental studies [10]. The entire life cycle ranges from 28 to 70 days, and starts when female wasps lay eggs into ticks through the penetration of their ovipositor into the tick’s body (Figure 1). After hatching, larvae (Figure 2) develop inside the tick. While no information is available about the pupal stage, adult wasps emerge from their tick hosts through a hole at the posterior end, with mating occurring soon after the emergence [9]. There have been no studies assessing the number of *Ixodiphagus* eggs released by females in natural conditions. However, based on experimental studies, it is estimated that during the entire life span, *I. hookeri* and *I. texanus* lay about 120 and 200 eggs, respectively [23,24].

Information about the preference for certain tick developmental stage remains unclear. For instance, some authors reported that larvae of *Ixodiphagus* are mostly detected in tick nymphs and adults when the latter are engorged, suggesting that parasitism is likely to occur in blood-fed ticks rather than in unfed ones [25]. However, an experimental study demonstrated that unfed nymphs of *I. ricinus* were more parasitized than other stages [10]. This observation was later confirmed with the finding of *I. hookeri* DNA in unfed *I. ricinus* nymphs collected from the environment [3]. Furthermore, it has been demonstrated that unfed ticks can be collected from vegetation, and after feeding them on laboratory animals (e.g., mice) the parasitoids emerge [26]. In their searching for ticks, *Ixodiphagus* spp. females may be driven by chemical attractants produced by vertebrate animals hosting ticks [10], as well as by tick feces [27]. In fact, some experiments have demonstrated that *I. hookeri* females appear to be attracted by odors produced by the haircoat of roe deer (*Capreolus capreolus*) and wild boar (*Sus scrofa*) [10] but not from those of mice, cattle, and rabbits [10]. This mechanism of attraction is crucial for facilitating the encounter of *Ixodiphagus* spp. with their preferred tick species [8], increasing the chances of completion of their lifecycle. Despite this observation, this is most likely not the general scenario in nature. It is believed that in most cases, hosts are attractive for ticks, in which eggs of the parasitoids are already present. The development of wasp larvae is directly dependent on nutrients contained in the engorged blood meal of the ticks; hence it is unlikely that *Ixodiphagus* larvae could develop in unfed ticks due to the depletion of nutrients [28]. This translates into a correlation between the occurrence of *Ixodiphagus* larvae, tick density, and infestation rate in vertebrate hosts [8,29]. For example, in *I. scapularis* nymphs the infestation of wasp parasitoids occurred only in individuals parasitizing white-tailed deer (*Odocoileus virginianus*) in the northeastern USA, and in areas with deer population density of 13–20 animals per km^2^ or higher [29]. In addition, no association was observed between the occurrence of wasps and *I. ricinus* infesting rodents in northern Europe [8], suggesting that the species of vertebrate host is crucial for the behavior of *Ixodiphagus* spp.. Despite the lack of an association between wasps and ticks of rodents, it is known that in laboratory conditions parasitoids develop and emerge from ticks that feed on mice. The dynamic of *Ixodiphagus* has been poorly assessed in field conditions. Based on the few studies conducted so far, adults fly for a short period of time. In Germany, adult wasps were found during 3–5 weeks, in late summer/early fall [10]. This seasonal activity overlaps with a high density and feeding activity of *I. ricinus* immature stages in the same area, which incidentally occurs when vertebrate hosts are also more abundant. For example, it has been demonstrated that wasps from ticks fed before July have a shorter developmental time compared with those from ticks engorged later on [10]. This finding is similar to those previously observed in field conditions in Texas (USA), where wasps required a development time of 25 and 33 days for ticks fed in May and September, respectively [9]. 

In southern Italy, the majority of ticks that tested positive for *I. hookeri* (i.e., 92%) were collected during fall–winter (from October to March) [3], when *I. ricinus* peaked [30]. Overall, the detection in ticks is related to developmental time of *Ixodiphagus* and to the synchronization with tick development [10]. Curiously, non-embryonated eggs of *I. hookeri* are able to survive over winter inside unfed nymphs of *I. ricinus* [31] and *I. scapularis* [6,25]. From a biological perspective, this characteristic allows wasp populations to survive through different seasons in spite of unfavorable climate conditions (e.g., extreme cold). 

The molecular detection of *Wolbachia* endosymbionts in *I. hookeri* [32] suggests that it could be the reason for the presence of *Wolbachia pipientis* in *I. ricinus* [33], with a role in their parthenogenesis (i.e., development from unfertilized eggs). This is demonstrated in other hymenopteran species (e.g., *Encarsia pergandiella*) [34]. Despite the suggested parthenogenesis for *Ixodiphagus* [24], the potential involvement of *Wolbachia* has never been demonstrated. Recently, the assessment of the microbiota in *I. ricinus* in high-throughput sequencing revealed the presence of a wide plethora of microorganisms, including *I. hookeri* and *Wolbachia* [35]. These multiple interactions among microorganisms in *I. ricinus* may affect a wasp population, influencing differences in its biology observed in different tick populations worldwide [10,36,37]. *Ixodiphagus* spp. have been widely reported in various species of ixodid ticks, with a broad distribution across all five inhabited continents [4,38,39], but more commonly reported from Europe and the US [3,6,29]. In fact, several hard tick species within the genera *Amblyomma*, *Dermacentor*, *Haemaphysalis*, *Hyalomma*, *Ixodes*, and *Rhipicephalus*, in various life stages, have been found parasitized by *Ixodiphagus* wasps (Table 1). So far, the only argasid soft tick found parasitized by an *Ixodiphagus* species (*I. mysorensis*) was *Ornithodoros* sp. [40].

## 3. Tick–Wasp Interaction in the Control of Ticks

Despite the existing body of literature describing the deadly interactions between ticks and wasps, the success of attempts conducted to control ticks by means of this parasitoid is still arguable. Historically, the use of *Ixodiphagus* spp. wasps to control tick populations dates back to 1908, when nymphs containing the parasitoid *I. hookeri* were shipped from Texas to South Africa, Portugal, and Italy [9]. Even with the emergence of *Ixodiphagus* adults from nymphs sent to South Africa, their use for tick control in this country failed. Unfortunately, *Ixodiphagus* nymphs sent to Portugal and Italy did not develop to the adult stage [9]. 

Later on, *Ixodiphagus* wasps were released in Massachusetts (USA) in various ways, (i) adults, (ii) inside *I. scapularis* nymphs, and iii) in mice infested by *I. scapularis* nymphs containing wasp larvae [75]. Interestingly, in 1927 mice and wild rabbits were trapped in the same area and presented a lower infestation rate by *D. variabilis* than years before (i.e., several hundred larvae and 30–40 nymphs per animal). Despite the apparent success in reducing the population of ticks, estimative methods of tick infestation were not used, making it difficult to correlate the reduction of tick population to wasp infestation [75]. Similarly, about 4,000,000 wasps were released from 1927 to 1932 in Montana, Colorado, Idaho, and Oregon (USA) to control *Dermacentor andersoni* ticks in the environment [76]. Albeit different methods of release (e.g., as adults, infected nymphs released on the grass, and infected nymphs parasitizing squirrels) had been employed, these attempts failed since the wasp population did not establish in the study area [11,76]. In fact, the recovery of parasitoids from ticks was successful only in Montana, where few adult wasps emerged from tick nymphs collected from squirrels [76]. 

From 1937 to 1939, approximately 90,000 *I. hookeri* females were released at Squibnocket Beach in Massachusetts, at two sites, a wild bog (in September and October 1937, and from April to July 1938) and a grassy hill area, close to the beach (in August 1938, and from May to August 1939) [12]. Despite the scarcity of immature ticks in the first location in the subsequent year (1938), adults remained abundant. In this area, a hurricane in September 1938 impaired continued observation. In subsequent tick estimations performed in 1940 and 1941, ticks were abundant but no *I. hookeri* were detected [12]. 

Similar attempts to control *I. ricinus* and *I. persulcatus* adults were conducted in Russia though the release of *I. hookeri* (n = 2600) adults, as well as larval and nymphal stages (n = 38,000) of *I. ricinus* containing wasp larvae [77]. In this case, low temperatures killed the wasps and the experiment failed [76]. Following the attempts above, the interest of the scientific community about *Ixodiphagus* spp. decreased, and this wasp was not investigated until the end of the last century, when a study conducted in Kenya demonstrated the potential of this parasitoid in controlling ticks [66]. In that study, approximately 150,000 *I. hookeri* specimens were released over a 1-year period to control *Amblyomma variegatum* in a field with 10 infested cattle. During the day, animals were kept in a grazing paddock (200 × 200 m), and at night in a resting area measuring 50 × 25 m. Wasps were released into the environment as adults and parasitizing *A. variegatum* nymphs. Monitoring was performed with monthly tick counting. Despite the reduction of the infestation rate of *A. variegatum* from 44 to 2 ticks per animal, population of *Rhipicephalus appendiculatus* did not vary at all [66], suggesting that this parasitoid is effective with a specific tick species. This putative specificity for some tick species should be considered in future studies focusing on the use of wasps for the control of ticks. 

## 4. Wasps in Ticks: Why Did the Control Attempts Fail?

Most of the attempts to control ticks through parasitoid wasps were carried out approximately 100 years ago, with limited information about the biology of this wasp and its interaction with ticks. Overall, these studies were mainly based on the limited knowledge regarding the ability of *Ixodiphagus* species to cause tick death. Important variables that may directly impact the use of wasps as an effective method for biological control of ticks remain unaddressed, including climatic conditions, target tick species, and density of vertebrate hosts. For instance, the first study conducted demonstrated a reduction in *I. scapularis* population parasitizing vertebrate animals in the subsequent year [75]. However, a long-term evaluation has never been conducted resulting in partial evidence. Indeed, these attempts were conducted through the release of wasps (i.e., adults or larvae inside ticks) directly into the environment, without the control of any factor that could have influenced the biology of the ticks or the wasps, or their survival and establishment [7,76,77]. Additionally, the strategies of releasing wasps (e.g., single or multiple releases) were not consistent across experiments, making direct comparisons difficult. 

The only successful *Ixodiphagus* field study, conducted in Kenya, provided detailed information about the tick population, method of release, measure of area where animals were kept, temperature (i.e., 24–31 °C), and targeted grazing areas [66]. These parameters suggested that the reduction of tick population was a result of the interactions with parasitoid wasps. Despite this promising outcome, this study was conducted on a small scale and no further follow-ups were performed to clarify the viability and sustainability of this control method on a long-term basis [66]. Additionally, the population of one tick species present, *R. appendiculatus,* did not reduce, suggesting a potential preference of *Ixodiphagus* species for some tick species [66]. 

All the compiled data indicate that tick–wasp interaction is an important factor for tick population balance in natural conditions. Nonetheless, the knowledge accumulated so far suggests that there are several limitations for the implementation of this control method under natural conditions. Both ticks and parasitoid wasps present complex biology and are heavily influenced by environmental conditions (e.g., temperature, rainfall, and air humidity), which poses additional challenges for its successful implementation for tick control. 

## 5. Gaps in the Knowledge and Future Perspectives

Despite *Ixodiphagus* having been considered for decades as a potential agent for the biological control of ticks, its biology and its interactions with ticks are not fully understood. Currently, little is known about its seasonality, development in different environmental conditions, synchrony with the life cycle of different tick species, and pupal stage. Therefore, studies including the assessment of tick–wasp seasonality in different geographic areas to evaluate the impact of this parasitoid in natural conditions are encouraged. In addition, the genetic characterization of different nuclear and mitochondrial genes of multiple species and isolates of *Ixodiphagus* is of interest for understanding the phylogenetic relationships among species, which will ultimately improve the knowledge on the evolutionary history of these wasps, and their association with ticks.

Future research using this parasitoid for biological tick control should carefully consider parameters such as targeted tick species based on biological understanding, density of vertebrate host(s) in the area, number of wasps, method of release, dimension of the area, climatic conditions, and seasonal patterns. Despite the need for searching for an effective alternative control strategy for tick balance in natural conditions, the use of this parasitoid for biological control of ticks is tough due to biological and environmental factors associated with ticks and these wasps.

## Figures and Tables

**Table 1:** Distribution of *Ixodiphagus* spp. parasitizing different tick species in the world.

Parasitoid	Tick	Tick Life Stage	Country	Reference
*I. texanus*	*H. leporispalustris*	Nymph	United States	[2]
*I. hookeri*	*R. sanguineus*	Nymph	United States	[41]
*I. hookeri*	*R. sanguineus*, *D. marginatus*	Nymph	United States	[9]
*I. hookeri*	*I. ricinus*	Nymph	France	[42]
*I. hookeri*	*H. concinna, D. reticulatus, D. venustus,* *R. sanguineus*	NA	France	[43]
*I. hookeri*	*R. sanguineus*	Nymph	Brazil	[44]
*I. hookeri*	*R. sanguineus*	NA	India	[45]
*I. hookeri*	*D. nitens*	NA	United States	[46]
*I. hookeri*	*D. variabilis*	NA	United States	[11]
*I. hookeri*	*H. aegyptium*	NA	South Africa	[47]
*I. hookeri*	*R. sanguineus*	Nymph	Nigeria	[48]
*I. hookeri*	*I. cookei*	Nymph	United States	[49]
*I. hookeri*	*R. sanguineus*	NA	United States	[50]
*I. texanus*	*H. leporispalustris*	Nymph	United States	[51]
*I. hookeri*	*R. sanguineus*	Nymph	United States	[52]
*I. mysorensis*	*Ornithodorus* sp.	NA	India	[40]
*I. texanus*	*I. persulcatus*	Nymph	Russia	[53]
*I. hookeri*	*I. ricinus*	Nymph	Czech Republic/Slovakia (Czechoslovakia)	[54]
*I. hookeri*	*R. sanguineus*	Nymph	Kenya	[55]
*I. hookeri*	*R. sanguineus*	Nymph	Africa	[56]
*Ixodiphagus* sp.	*H. bancrofti, H. bremneri, I. holocyclus, I. tasmani*	NA	Australia	[57]
*I. hookeri*	*R. sanguineus*	NA	Indonesia	[58]
*I. hookeri*	*R. sanguineus*	Nymph	Malaysia	[59]
*I. texanus*	*H. leporispalustris*	Larva, Nymph	Canada	[60]
*I. hookeri*	*I. dammini*	Nymph	United States	[21]
*I. hookeri*	*H. punctata*	Nymph	Spain	[61]
*I. hookeri*	*A. variegatum*	Nymph	Kenya	[62]
*I. hookeri*	*I. ricinus*	NA	France	[63]
*I. texanus*	*I. dammini*	Nymph	United States	[64]
*I. hookeri*	*R. sanguineus*	Nymph	Mexico	[65]
*I. hookeri*	*I. scapularis*	Nymph	United States	[66]
*I. hookeri*	*I. scapularis*	Nymph	United States	[25]
*I. hookeri*	*A. variegatum*	Nymph	Kenya	[67]
*I. hookeri*	*I. scapularis*	Nymph	United States	[29]
*I. hookeri*	*R. sanguineus*	Nymph	Venezuela	[68]
*I. hookeri*	*A. variegatum*	Nymph	Kenya	[37]
*I.* *hookeri*	*H. concinna*	Nymph	Slovakia	[26]
*I. taiaroaensis*	*I. uriae, I. eudyptidis*	Larva, Nymph	New Zealand	[69]
*I. hookeri*	*I. ricinus*	Nymph	Germany	[10]
*I. hookeri*	*I. ricinus*	Nymph	Netherlands	[32]
*I. hookeri, I. texanus*	*R. sanguineus, Amblyomma* sp.	Nymph	Brazil	[70]
*I. hookeri*	*I. ricinus*	Nymph	France	[32]
*I. hookeri, I. texanus*	*R. sanguineus*	Nymph	Panama	[71]
*I. hookeri*	*I. ricinus*	Nymph, Adult	Italy	[3]
*I. hookeri*	*I. ricinus*	Nymph	Slovakia	[22]
*Ixodiphagus* sp.	*R. sanguineus*	Nymph, Adult	Brazil	[4]
*I. hookeri*	*I. ricinus*	Nymph	Finland	[5]
*I. hookeri*	*R. sanguineus*	Nymph	United States	[72]
*I. hookeri*	*R. microplus*, *I. persulcatus*, *D. silvarum*, *H. concinna*	Adult	Côte d’Ivoire, Senegal, Russia	[39]
*I. hookeri*	*I. ricinus*	Larva, Nymph	Netherlands	[8]
*I. hookeri*	*I. ricinus*, *H. concinna*	Nymph	Slovakia	[1]
*I. hookeri*	*I. ricinus*	Nymph	France	[35]
*I. hookeri*	*I. ricinus*	Nymph	United Kingdom	[73]
*I. hookeri*	*A. nodosum*	Nymph, Adult	Brazil	[74]
*I. hookeri*	*I. ricinus*	Nymph	Hungary	[6]

NA: Not available.

## Data Availability

Not applicable.
